# Vitamin D intake is associated with insulin sensitivity in African American, but not European American, women

**DOI:** 10.1186/1743-7075-7-28

**Published:** 2010-04-14

**Authors:** Jessica A Alvarez, Nikki C Bush, Suzanne S Choquette, Gary R Hunter, Betty E Darnell, Robert A Oster, Barbara A Gower

**Affiliations:** 1 Department of Nutrition Sciences, University of Alabama at Birmingham, 1625 University Blvd, Birmingham, Alabama, 35294, USA; 2 Center for Clinical and Translational Science, University of Alabama at Birmingham, JT 1502, 619 19th Street S, Birmingham, Alabama, 35249, USA; 3 Department of Human Studies, University of Alabama at Birmingham, 901 13th Street S, Birmingham, Alabama, 35294, USA; 4 Division of Preventative Medicine, University of Alabama at Birmingham, 1717 11th Avenue S, Birmingham, Alabama, 35205, USA

## Abstract

**Background:**

The prevalence of type 2 diabetes is higher among African Americans (AA) vs European Americans (EA), independent of obesity and other known confounders. Although the reason for this disparity is not known, it is possible that relatively low levels of vitamin D among AA may contribute, as vitamin D has been positively associated with insulin sensitivity in some studies. The objective of this study was to test the hypothesis that dietary vitamin D would be associated with a robust measure of insulin sensitivity in AA and EA women.

**Methods:**

Subjects were 115 African American (AA) and 137 European American (EA) healthy, premenopausal women. Dietary intake was determined with 4-day food records; the insulin sensitivity index (S_I_) with a frequently-sampled intravenous glucose tolerance test and minimal modeling; the Homeostasis Model Assessment of Insulin Resistance (HOMA-IR) with fasting insulin and glucose; and body composition with dual-energy X-ray absorptiometry.

**Results:**

Vitamin D intake was positively associated with S_I _(standardized β = 0.18, *P *= 0.05) and inversely associated with HOMA-IR (standardized β = -0.26, *P *= 0.007) in AA, and the relationships were independent of age, total body fat, energy intake, and % kcal from fat. Vitamin D intake was not significantly associated with indices of insulin sensitivity/resistance in EA (standardized β = 0.03, *P *= 0.74 and standardized β = 0.02, *P *= 0.85 for S_I _and HOMA-IR, respectively). Similar to vitamin D, dietary calcium was associated with S_I _and HOMA-IR among AA but not EA.

**Conclusions:**

This study provides novel findings that dietary vitamin D and calcium were independently associated with insulin sensitivity in AA, but not EA. Promotion of these nutrients in the diet may reduce health disparities in type 2 diabetes risk among AA, although longitudinal and intervention studies are required.

## Background

Epidemiological research suggests that low vitamin D intake is associated with greater risk of type 2 diabetes [1]. Vitamin D may reduce disease risk by promoting insulin sensitivity, as suggested by *in vitro *studies and associations of 25-hydroxyvitamin D (25(OH)D) with insulin sensitivity [2,3]. Supplementation trials, however, have resulted in mixed outcomes, likely due to differences in the populations and varying measurements of insulin sensitivity [4]. To our knowledge, no previous study has investigated whether dietary vitamin D is related to insulin sensitivity.

African Americans (AA) have lower insulin sensitivity and, subsequently, a greater risk of type 2 diabetes compared to European Americans (EA) [5], although the cause of this disparity is multi-factorial and not completely understood. Ethnic differences also exist in vitamin D status such that AA have lower circulating 25(OH)D and lower intakes of vitamin D than EA [6,7]. Whether ethnic differences in vitamin D status contribute to the ethnic disparities in type 2 diabetes is not clear.

Ethnic differences may also exist in the relationship of vitamin D with insulin sensitivity. Analysis of the Third National Health and Nutrition Examination Survey (NHANES 1988-1994) revealed inverse associations between serum 25(OH)D and the Homeostasis Model Assessment of Insulin Resistance (HOMA-IR) in EA and Mexican Americans but not AA [8]. However, indices derived from fasting insulin and glucose values, such as HOMA-IR, primarily reflect hepatic insulin resistance [9], and the accuracy of HOMA-IR in predicting whole-body insulin sensitivity differs among AA and EA [10]. Thus it is not clear if ethnic differences would exist when a robust measure of whole-body insulin sensitivity is used. Further, whether an ethnic difference exists in the relationship of dietary vitamin D with insulin sensitivity has not been investigated.

The objective of this study was to examine the relationship of dietary vitamin D with insulin sensitivity in healthy AA and EA women using both a robust measure of insulin sensitivity, the frequently-sampled intravenous glucose tolerance test (FSIGT), and a commonly used surrogate of whole body insulin resistance, HOMA-IR. We tested the hypotheses that: 1) dietary vitamin D is positively associated with insulin sensitivity, independent of adiposity and total energy intake, and 2) the relationship of dietary vitamin D with insulin sensitivity differs between AA and EA. As calcium has also been shown to be associated with insulin sensitivity [11] and to have ethnic differences in its relationship with insulin sensitivity [12], we also examined associations of dietary calcium with insulin sensitivity in AA and EA women.

## Methods

### Subjects

Subjects were 252 healthy, premenopausal women (115 AA and 137 EA) who enrolled in one of two studies at the University of Alabama at Birmingham (UAB) investigating the role of metabolic factors in the etiology of obesity. This sub-study uses baseline data collected prior to intervention (where relevant) and includes only subjects who completed a baseline FSIGT and diet records. Subjects were recruited through word-of-mouth or newspaper advertisements, and baseline data were collected between 1995 and 2005. All women were non-smokers, sedentary (defined as < 2 hrs per week of exercise within the last yr), consumed < 400 g of alcohol per week, had a history of regular menstrual cycles, had normal glucose tolerance, and were not taking medications known to affect body composition or metabolism. Subject BMI ranges were ≤ 25 kg/m^2 ^or 27-30 kg/m^2^, depending on the protocol. Ethnicity was self-defined with both parents and all immediate grandparents required to be of the same ethnic group. All research for this study was in compliance with the Helsinki Declaration and was approved by the UAB Institutional Review Board for Human use. Informed consent for all procedures was obtained upon recruitment.

### Protocol

Before metabolic testing, subjects completed a 4-wk weight stabilization period with weights recorded 3 times weekly and food provided by the General Clinical Research Center (GCRC) during the last 2 weeks to ensure weight maintenance and a consistent macronutrient intake. Four-day food records were completed prior to the weight stabilization period. Insulin sensitivity and body composition testing were assessed at the GCRC during an overnight inpatient stay following weight stabilization and while subjects were in the follicular phase of the menstrual cycle.

### Insulin Sensitivity Testing

The insulin sensitivity index (S_I_) and HOMA-IR were investigated as indices of insulin sensitivity/resistance. S_I_, an index of whole-body insulin sensitivity [13], was obtained with either a tolbutamide-modified FSIGT or an insulin-modified FSIGT, as previously described [14,15]. To perform either test, flexible intravenous catheters were placed in the antecubital spaces of both arms after a 12-hr overnight fast. The average of three blood samples collected over a 20-min period was used for assessment of fasting glucose and insulin. Glucose was then intravenously administered (50% dextrose, 11.4 g/m^2^) at time "0" and multiple blood samples were collected from times "2 min" through "180 min" relative to glucose administration. A tolbutamide (125 mg/m^2^) or insulin (0.02 units/kg) bolus was injected at time "20 min." Sera were stored at -85°C until analysis for glucose and insulin, values for which subsequently were entered into the MINMOD computer program (version 3.0,^© ^Richard N. Bergman) for determination of S_I _[16]. The change in FSIGT protocol (tolbutamide vs. insulin-modified) occurred due to the discontinuation of tolbutamide by the manufacturer. As S_I _values measured using the insulin-modified FSIGT are lower than those obtained with the tolbutamide-modified FSIGT [17], "test type" was included as a variable in multiple linear regression models to account for differences in the tests. HOMA-IR was calculated as [fasting insulin (μU/ml) × fasting glucose (mmol/L)]/22.5. The S_I _value of one woman was not available due to procedural issues, although fasting values were not affected.

### Dietary Intake

A registered dietitian trained subjects to complete 4-day food records, including 2 weekdays and 2 weekend days. Food records were analyzed using the Nutrition Data System for Research (NDS-R) software (Nutrition Coordinating Center, University of Minnesota, MN, Database versions 4.04_32 and 4.05_33, release date 2001-2002). Food records were analyzed for total kilocalories (kcal), % kcal from carbohydrate, fat, and protein, and calcium (mg/d), and vitamin D (IU/d). Only records with at least 3 of the 4 days completed were used.

### Body Composition

Subjects were weighed to the nearest 0.1 kg (Scale-tronix 6702W; Scale-tronix, Carol Stream, IL) in minimal clothing without shoes, and height was measured without shoes using a digital stadiometer (Heightronic 235; Measurement Concepts, Snoqualmie, WA). Total body fat and lean body mass were measured by dual-energy X-ray absorptiometry (DXA) using either a GE Lunar Prodigy densitometer or a Lunar DPX-L densitometer (GE LUNAR Radiation Corp., Madison, WI) with subjects lying flat on their backs with their arms to the sides and in light clothing. Results obtained with these instruments differ by 4% or less [18] and are accounted for by the addition of "test type" to regression analyses. DXA data were available for 114 AA and 135 EA.

### Glucose and Insulin Analysis

Serum glucose and insulin were analyzed in the Core Laboratory of the GCRC and the Clinical Nutrition Research Center at UAB. Glucose was measured in 10 μl sera with an Ektachem DT II System (Johnson and Johnson Clinical Diagnostics, Rochester, NY). In the Core Laboratory, this analysis has a mean intra-assay and inter-assay coefficient of variation (c.v.) of 0.61% and 1.45%, respectively. In one study, insulin was assayed in duplicate 200 μl aliquots with Diagnostics Products Corporation "Coat-A-Count" kits (Los Angeles, CA). In the Core Laboratory, this assay has a sensitivity of 1.9 μIU/ml, a mean intra-assay c.v. of 5%, and a mean inter-assay c.v. of 6%. In the other study, insulin was assayed in duplicate 100 μl aliquots using double-antibody radioimmunoassay (Linco Research, St. Charles, MO) reagents. In the Core Laboratory, this assay has a sensitivity of 3.35 μIU/ml, a mean intra-assay c.v. of 3.49%, and a mean inter-assay c.v. of 5.57%. The difference in insulin assays is accounted for by the addition of "test type" to regression analyses.

### Statistical Analyses

Means and standard deviations for all variables of interest were computed among all subjects and within each ethnic group. Two-group t-tests were used to compare continuous variables between ethnic groups. Ethnic group comparisons of subjects tested with an insulin-modified FSIGT and the Lunar DPX-L DXA machine (indicated in analyses as "test type 1") were analyzed with the two-group chi-square test. Pearson correlation analysis was used to examine the relationships of dietary vitamin D and calcium with fasting glucose, fasting insulin, HOMA-IR, and S_I_. Multiple linear regression analyses were used to determine if dietary calcium and/or vitamin D intake was independently related to S_I _and HOMA-IR after adjusting for test type, age, ethnic group, total kcal intake, % kcal from fat, and total body fat. These statistical analyses were performed among all subjects and separately by ethnic group. The distributions for S_I_, vitamin D, calcium, HOMA-IR, fasting glucose, and fasting insulin deviated from a normal distribution and were thus log_10_-transformed. Statistical analyses were performed using the SAS software package (version 9.1; SAS Institute, Cary, NC), and statistical tests were 2-sided and assumed a 5% significance level.

## Results

Subject clinical characteristics and reported dietary intakes by ethnicity are detailed in Table 1. AA and EA subjects were similar in age, anthropometrics, and body composition. Fasting glucose and S_I _were lower in AA vs. EA (*P *< 0.001). Dietary analyses indicated that AA reported consuming less total kcal (*P *= 0.02), % kcal from carbohydrate (*P *= 0.03), vitamin D (*P *= 0.01), and calcium (*P *< 0.001), and greater % kcal from fat (*P *= 0.03). The proportion of subjects being tested with insulin-modified FSIGT and the Lunar DPX-L DXA machine did not differ significantly by race (AA 77%, EA 71%, *P *= 0.24).

**Table 1:** Clinical and dietary variables in all subjects combined and by ethnic group

	All (n = 252)	AA (n = 115)	EA(n = 137)
Age (yr)	34.5 ± 6.0	34.0 ± 6.1	34.8 ± 6.0
Weight (kg)	75.3 ± 8.8	74.5 ± 9.0	76.0 ± 8.6
Height (cm)	164.5 ± 6.5	163.6 ± 6.6	165.2 ± 6.3*
BMI (kg/m^2^)	27.7 ± 2.3	27.8 ± 2.4	27.7 ± 2.2
Total body fat (kg)	31.4 ± 6.6	32.0 ± 6.4	30.6 ± 6.4
Lean body mass (kg)	40.3 ± 4.1	40.3 ± 4.5	40.4 ± 3.8
Fasting glucose (mmol/l)^‡^	4.9 ± 0.3	4.8 ± 0.3	5.0 ± 0.3^†^
Fasting insulin (pmol/l)^‡^	77.8 ± 29.9	82.0 ± 35.4	75.0 ± 24.3
HOMA-IR^‡^	2.5 ± 1.0	2.6 ± 1.2	2.4 ± 0.8
S_I _[× 10^-4^min^-1^/(μIU/ml)]	3.6 ± 2.8	2.8 ± 2.0	4.3 ± 3.2^†^
Energy intake (kcal)	1767.2 ± 474.9	1687.2 ± 485.0	1834.3 ± 457.2*
% kcal from carbohydrates	48.1 ± 8.4	46.8 ± 8.8	49.1 ± 7.9*
% kcal from fat	36.8 ± 6.6	37.8 ± 6.8	35.9 ± 6.4*
% kcal from protein	15.5 ± 3.1	15.9 ± 3.1	15.3 ± 3.2
Vitamin D (IU/d)	125.5 ± 83.6	111.5 ± 74.8	133.7 ± 88.9*
Calcium (mg/d)	628.3 ± 279.5	507.9 ± 205.2	729.3 ± 293.8^†^

Data are presented as mean ± SD. Abbreviations: AA, African American; EA, European American; HOMA-IR, homeostasis model assessment of insulin resistance; S_I_, insulin sensitivity index. Statistical analyses were performed on log_10_-transformed data for S_I_, vitamin D, calcium, HOMA-IR, fasting glucose, and fasting insulin. P for difference between groups determined using a two-group t-test.

*P < 0.05 for difference between ethnic groups

^†^*P *< 0.001 for difference between ethnic groups

^‡^n = 114 AA

Pearson correlation coefficients for dietary vitamin D and calcium with indices of insulin sensitivity/resistance are reported in Table 2. Among all subjects, dietary vitamin D was significantly, positively associated with S_I _(*P *= 0.01), and there was a trend towards a significant inverse association between vitamin D intake and HOMA-IR and fasting insulin (*P *= 0.08 for both). Dietary calcium was significantly, positively associated with S_I _(*P *< 0.001). In multiple linear regression analyses among all subjects, after adjustment for ethnicity, test type, age, total body fat, energy intake, and % kcal from fat, a trend towards a significant, positive association between dietary vitamin D and S_I _remained (standardized β = 0.11, *P *= 0.07), and dietary vitamin D was significantly, inversely associated with HOMA-IR (standardized β = -0.15, *P *= 0.03). Similarly, among all subjects, there was a trend towards a significant independent relationship between dietary calcium and S_I _(standardized β = 0.13, *P *= 0.09), and dietary calcium was independently, inversely associated with HOMA-IR (standardized β = -0.17, *P *= 0.04). If both dietary vitamin D and calcium were added to the models as independent variables, neither nutrient was significantly associated with S_I _(*P *= 0.25 and 0.34, respectively) or HOMA-IR (*P *= 0.18 and 0.25, respectively).

**Table 2:** Pearson correlations of dietary vitamin D and calcium with measures of insulin and glucose metabolism

	Vitamin D			Calcium		
	All	AA	EA	All	AA	EA
Fasting glucose	-0.03	-0.13	-0.01	-0.001	-0.07	-0.12
Fasting insulin	-0.11*	-0.20^†^	0.01	-0.08	-0.10	-0.03
HOMA-IR	-0.11*	-0.21^†^	0.01	-0.08	-0.10	-0.05
S_I_	0.15^†^	0.17*	0.06	0.26^‡^	0.15	0.13

Pearson correlation coefficient (r) is reported. Abbreviations: FSIGT, frequently-sampled intravenous glucose tolerance test; AA, African American; EA, European American; HOMA-IR, homeostasis model assessment of insulin resistance; S_I_, insulin sensitivity index. Statistical analyses were performed on log_10_-transformed data for S_I_, vitamin D, calcium, HOMA-IR, fasting glucose, and fasting insulin.

*P = 0.05-0.10

^†^*P *< 0.05

^‡^*P *< 0.001

### Analyses by ethnic group

Correlation analyses by ethnic group, as reported in Table 2, revealed a trend towards a significant, positive association between dietary vitamin D and S_I _(*P *= 0.07) and significant, inverse associations of dietary vitamin D with HOMA-IR and fasting insulin (*P *= 0.02 and 0.03, respectively) and among AA. Dietary vitamin D was not significantly associated with any measure of insulin action in EA, and dietary calcium was not significantly associated with any measure of insulin action in either EA or AA.

**Figure 1:**
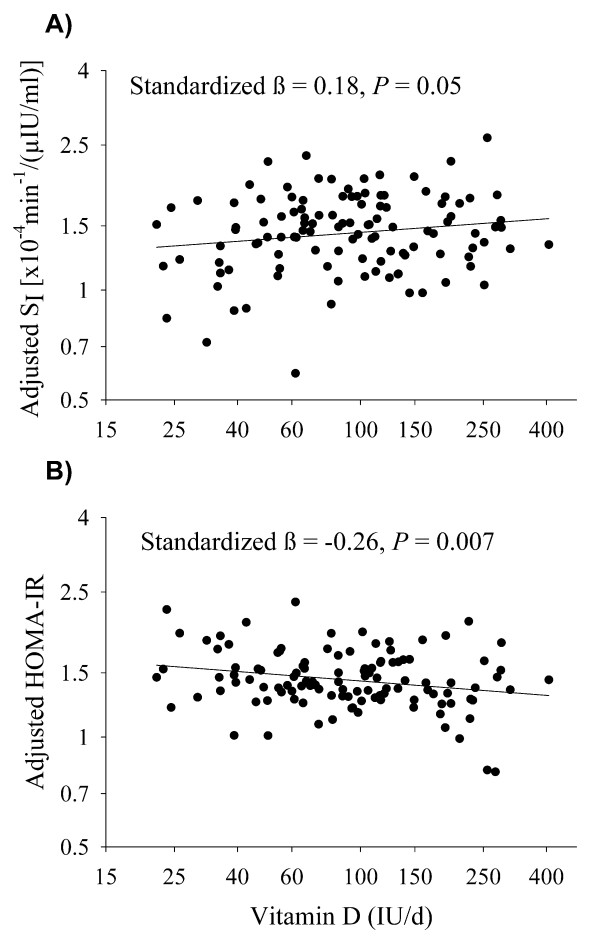
**Independent relationship between vitamin D intake and insulin sensitivity in African Americans**. A) Vitamin D vs. Insulin Sensitivity Index (S_I_) and B) Vitamin D vs. Homeostasis Model Assessment of Insulin Resistance (HOMA-IR). Relationships are adjusted for age, kcal intake, % kcal from fat, total body fat, and test type. The relationships among European Americans was not statistically significant (standardized β = 0.03, *P *= 0.74 and standardized β = 0.02, *P *= 0.85 for S_I _and HOMA-IR, respectively).

In MLR analysis by ethnic group, the positive association between dietary vitamin D and S_I _among AA remained after adjustment for test type, age, total body fat, energy intake, and % kcal from fat (Figure 1A), and dietary vitamin D remained significantly, inversely associated with HOMA-IR in AA (Figure 1B). Dietary vitamin D was not significantly associated with S_I _or HOMA-IR in EA. Among AA, the independent relationship between dietary calcium and S_I _was not significant (*P *= 0.16), although dietary calcium was significantly, inversely associated with HOMA-IR (standardized β = -0.23, *P *= 0.047). Dietary calcium was not significantly associated with S_I _or HOMA-IR in EA (*P *= 0.31 and *P *= 0.57 for S_I _and HOMA-IR, respectively). Among AA, if both dietary vitamin D and calcium were added to the MLR models as independent variables, only vitamin D emerged as a statistically significant correlate of HOMA-IR (*P *= 0.046), whereas neither nutrient was independently associated with S_I _(*P *= 0.15 and 0.58, respectively). Neither vitamin D nor calcium was significantly associated with S_I _(*P *= 0.86 and 0.33, respectively) or HOMA-IR (*P *= 0.61 and 0.46, respectively) when both nutrients were added to the MLR models for EA.

## Discussion

The objective of this study was to examine the relationships of dietary vitamin D with both whole-body insulin sensitivity derived from a FSIGT and HOMA-IR, a surrogate measure of whole-body insulin sensitivity. Additionally, we sought to determine if the relationships of dietary vitamin D with insulin sensitivity differed by ethnicity. Results indicated that, in this sample of women, both dietary vitamin D and calcium were positively associated with insulin sensitivity. However, the relationships were specific for AA women.

To our knowledge, this is the first study to investigate associations of dietary vitamin D with insulin sensitivity. In our sample, dietary vitamin D was positively associated with S_I _and inversely associated HOMA-IR, independent of strong determinants of insulin sensitivity. Post-hoc comparisons of the relationships of vitamin D with S_I _and HOMA-IR indicated that dietary vitamin D was more strongly associated with HOMA-IR than S_I _(*P *= 0.02 determined from POWCOR program [19]). As HOMA-IR primarily reflects hepatic insulin resistance [9] and S_I _captures both hepatic and peripheral insulin sensitivity [13], dietary vitamin D may be more related to hepatic insulin sensitivity than peripheral insulin sensitivity.

In analyses by ethnicity, we found dietary vitamin D to be positively associated with insulin sensitivity in AA but not EA, indicative of an ethnic difference in the relationship of dietary vitamin D with insulin sensitivity. It is not clear why the relationship between dietary vitamin D and insulin sensitivity would be specific for AA. An ethnic difference in the physiological effects of oral calcitriol has been noted in that calcitriol increased calcium absorption to a lesser degree in AA vs. EA premenopausal women [20]. Given the superior calcium economy of AA, as evidenced by resistance to the bone-resorbing effects of parathyroid hormone and greater renal calcium conservation [6], it is plausible that dietary vitamin D effects are directed to non-skeletal processes in AA to a greater extent than in EA. Vitamin D intervention studies comparing responses in both AA and EA populations are required to confirm the ethnic differences.

No other study, to our knowledge, has examined the role of ethnicity in determining associations between dietary vitamin D and S_I_, although Scragg et al. [8] reported that circulating 25(OH)D was inversely associated with HOMA-IR in EA but not AA. This finding in conjunction with ours indicates that among AA dietary vitamin D may have differential effects on insulin sensitivity than endogenously produced vitamin D, the major component of serum 25(OH)D. Dietary vitamin D typically comprises only about 10-20% of circulating vitamin D [21]. However, considering the limited production of endogenous vitamin D in AA [22], dietary vitamin D may be more important in this ethnic group with regards to insulin sensitivity. Studies investigating all forms and sources of vitamin D, including serum 25(OH)D, and their relationships with robust measures of insulin sensitivity, such as a FSIGT, in multiple ethnic groups are required.

Analyses using dietary calcium mirrored results of dietary vitamin D. Previous studies that have reported a positive association of dietary calcium with insulin sensitivity have not included vitamin D in analyses [11]. In addition to sharing some of the same food sources (e.g, fortified milk products and breakfast cereals), both nutrients are involved in regulating intracellular calcium fluxes which, in turn, are a necessary mediator of insulin action [3]. Thus it is difficult to distinguish the influences of one nutrient from the other. On the other hand, vitamin D has been shown to have direct effects on promoting insulin signaling *in vitro *[2], and our results showed that, among AA, vitamin D but not calcium was significantly associated with HOMA-IR when both nutrients were added as independent variables in MLR analyses. These results suggest that the relationship of vitamin D with insulin sensitivity, at least in AA, is independent of calcium. Further study is needed to determine the independent influences of these nutrients on insulin sensitivity, as well as the potential additive effects of vitamin D and calcium, as has been previously noted [1].

Although the mean intake of both ethnic groups was below the current Dietary Reference Intakes for vitamin D and calcium [23], AA reported less vitamin D and calcium intake than EA. This ethnic difference is consistent with NHANES reports [7,24]. AA are at greater risk for vitamin D deficiency, not only because of lower vitamin D intakes, but also due to reduced cutaneous vitamin D_3 _synthesis secondary to greater melanin pigmentation [22]. Given the ethnic disparity of lower insulin sensitivity and greater prevalence of type 2 diabetes among AA, our finding of beneficial associations of dietary vitamin D and calcium with insulin sensitivity in this ethnic group has important clinical and public health implications and supports the promotion of efforts to increase consumption of vitamin D and calcium-containing foods among AA.

The major strengths of this study included the use of robust measures of insulin sensitivity and body composition. In addition, our ethnic groups were relatively homogenous in body composition, thus allowing us to eliminate variations in body composition as potential confounders. Since the analyses of the food records were performed, updated data on the vitamin D content of foods have become available [25], thus it is likely that the reported vitamin D intake is underestimated with the NDS-R version used. However, assuming there is no biasing effect on the amount underestimated, the relationships observed should not be affected. The use of food records is inherently limited due to the tendency of subjects to under-report food intake; however, doubly-labeled water analysis in a sub-set of subjects revealed that both ethnicities under-reported food intake to a statistically similar degree (data not shown). Furthermore, the finding of statistically significant results would suggest that more accurate determinants of vitamin D and calcium intake would only strengthen our findings. Although general vitamin and mineral supplement use was not associated with S_I _and did not differ by ethnic group (data not shown), we could not differentiate whether subjects specifically consumed vitamin D and calcium supplements. In addition, biochemical measures of calcium or vitamin D intake/absorption (i.e, serum/urinary calcium or serum 25(OH)D) were not available. As findings could be related to other unmeasured factors associated with high vitamin D and calcium intake, future studies will need to confirm our results using not only measures of dietary intake, but taking into account specific dietary supplement intake, serum 25(OH)D, and serum/urinary calcium. Further, the cross-sectional nature of the study limits our ability to infer cause-effect relationships, and our results may not be generalizable to groups other than AA and EA premenopausal women.

## Conclusions

In conclusion, dietary vitamin D and calcium were positively associated with insulin sensitivity in premenopausal AA but not EA women. Our findings suggest that promoting dietary vitamin D and calcium intake may be a practical means of reducing type 2 diabetes risk among AA. Further studies are required to confirm an insulin-sensitizing effect of vitamin D and calcium in other populations, to determine whether such effects are hepatic or peripheral, to identify independent effects of vitamin D and calcium, and to determine mechanisms mediating potential ethnic differences in the relationship of dietary vitamin D and calcium with insulin sensitivity.

## Competing interests

The authors declare that they have no competing interests.

## Authors' contributions

JA participated in the study design and development of hypotheses, acquisition of the data, statistical analyses, interpretation of results, and initial and final drafting of the manuscript. NB and SC contributed to the study design and development of hypotheses, acquisition of the data, and drafting of the manuscript. GH contributed to the study design, acquisition of the data, interpretation of results, and critical review of the manuscript. BD contributed to acquisition of the data and provided critical review of the manuscript. RO assisted in statistical analyses and provided critical review of the manuscript. BG participated in the study design and development of hypotheses, acquisition of the data, interpretation of results, and drafting and critical review of manuscript. All authors reviewed and approved the final manuscript.
